# Discovering coherency of specific gene expression and optical reflectance properties of barley genotypes differing for resistance reactions against powdery mildew

**DOI:** 10.1371/journal.pone.0213291

**Published:** 2019-03-19

**Authors:** Matheus Thomas Kuska, Jan Behmann, Mahsa Namini, Erich-Christian Oerke, Ulrike Steiner, Anne-Katrin Mahlein

**Affiliations:** 1 Institute for Crop Science and Resource Conservation (INRES) - Plant Diseases and Plant Protection, University of Bonn, Bonn, Germany; 2 Institute of Sugar Beet Research (IfZ), Göttingen, Germany; Universita degli Studi di Pisa, ITALY

## Abstract

Hyperspectral imaging has proved its potential for evaluating complex plant-pathogen interactions. However, a closer link of the spectral signatures and genotypic characteristics remains elusive. Here, we show relation between gene expression profiles and specific wavebands from reflectance during three barley—powdery mildew interactions. Significant synergistic effects between the hyperspectral signal and the corresponding gene activities has been shown using the linear discriminant analysis (LDA). Combining the data sets of hyperspectral signatures and gene expression profiles allowed a more precise differentiation of the three investigated barley-*Bgh* interactions independent from the time after inoculation. This shows significant synergistic effects between the hyperspectral signal and the corresponding gene activities. To analyze this coherency between spectral reflectance and seven different gene expression profiles, relevant wavelength bands and reflectance intensities for each gene were computed using the Relief algorithm. Instancing, xylanase activity was indicated by relevant wavelengths around 710 nm, which are characterized by leaf and cell structures. *HvRuBisCO* activity underlines relevant wavebands in the green and red range, elucidating the coherency of *RuBisCO* to the photosynthesis apparatus and in the NIR range due to the influence of *RuBisCO* on barley leaf cell development. These findings provide the first insights to links between gene expression and spectral reflectance that can be used for an efficient non-invasive phenotyping of plant resistance and enables new insights into plant-pathogen interactions.

## Introduction

Molecular analysis entered as a rapid and advanced method for pre-selection and resistance screenings in plant breeding processes [1]. However, it is necessary to test the function of the genome of breeding material in greenhouse and field trials to assess their stability in different environments [2]. In addition, changes in gene expression and protein synthesis change the metabolism which influences the plant phenotype [3]. Phenotyping by visual estimation is labor and cost-intensive [4]. To overcome this bottleneck, many recent investigations deal with optical sensor approaches for a non-invasive and efficient evaluation of plant properties [5, 6]. Within this context, hyperspectral imaging (HSI) is a promising tool to assess different plant parameters with high accuracy [4]. Compared to conventional red, green, blue (RGB) cameras, HSI includes high resolution optical techniques with increased spectral resolution. HSI assesses narrow wavebands in the visual light from 400–700 nm (VIS), in the near-infrared from 700–1000 nm (NIR), and in the shortwave infrared from 1000–2500 nm (SWIR). Different parameters of plant physiology, chemistry and health status can be derived from the electromagnetic spectrum with a range of 400–2500 nm [7, 8]. This enables a non-invasive detection and characterization of fungal plant pathogens as well as plant resistance reactions by hyperspectral imaging [9–11]. Hyperspectral imaging data are often analyzed and interpreted with histological and physiological observations as well as information from established sensors such as chlorophyll fluorescence [8, 9]. The correlation of genes or proteins to spectral reflectance patterns has not yet proven, despite the fact that many plant resistance reactions and the plant immunity pathways are known on the omic level [12].

In the present study, gene expression profiles were linked to hyperspectral reflectance signatures. To elucidate functional correlations and benefits for plant resistance breeding, *Hordeum vulgare* L. and *Blumeria graminis* f.sp. *hordei* (*Bgh*) was used as a model system. Besides a susceptible barley genotype (WT), two near-isogenic lines of *H*. *vulgare* cv. Ingrid M.C. 20 (mildew locus o 3 (*mlo3*) based resistance) and cv. Pallas 01 (Mildew locus a 1 and 12 (*Mla1*; *Mla12*) based resistance) were used to analyze different plant-pathogen interactions. The *mlo* dysfunction allows the induction of effective cell wall appositions (CWAs—papillae) that developed at the penetration site of *Bgh* during the first ~40 hours after inoculation (hai) and inhibit the penetration [13]. The *Mla* based resistance is characterized by fast local or single-cell hypersensitive responses (HR) against *Bgh* [14].

Expression of seven genes that are indicators for plant resistance, cell signaling and cell metabolism were analyzed 0, 12, 24, 48 and 72 hai. As resistance indicators, three different *PR* genes were analyzed. The *PR2* transcript encodes a *ß*-1,3-glucanase, which has high similarity to a putative, extracellular localized *ß*-1,3-endo glucosidase [15]. This enzyme hydrolyzes (1–3, 1–6) branched *ß*-glucans of fungal cell walls. A second protein encoded against fungal pathogens is the *PR3* expressed transcript, encoding a chitinase class 2 (EC 3.2.1.14) [16]. This chitinase class is generally described as acidic and extracellular, but detectable in the apoplast and protoplast, respectively [17]. Further important stress correlated genes correspond to the *PR5* family [18]. *PR5* expression encodes a thaumatin-like protein, with several putative properties e.g. antifungal activity and cell regulation during abiotic and biotic stress [18, 19]. Jasmonic acid is a further important resistance and cell signaling hormone [20]. It is derived from the cell membrane associated linoleic acid and induce *de novo* synthesis of jasmonate induced proteins (JIP) [21]. During fungal pathogenesis the plant metabolism is also influenced by plant sugars, which can regulate the gene expressions e.g. of ribulose-1,5-bisphosphate carboxylase small subunit (*RuBisCO*) [22, 23]. Thereby, abscisic acid as an important plant hormone for plant development is also influenced [24]. Abscisic acid is responsible for regulation of gene expression during stress responses and natural senescence procedures. In barley the dehydration-responsive factor 1 (*HvDRF1*) is involved in abscisic acid activation and mediated gene regulation [25]. Until now, important regulators on the omics level during a pathogen infestation have not been considered in plant phenotyping studies using hyperspectral imaging.

Here, an applied linear discriminant analysis (LDA) revealed significant synergistic effects between the hyperspectral signal and the corresponding gene activities during early barley-*Bgh* interactions. The Relief algorithm determined relevant wavelengths to distinguish between *Bgh* inoculated and healthy barley and to characterize relevant spectral wavelengths for gene expression profiles.

## Material and methods

### Plant material and cultivation

Plants were grown in commercial substrate (Klasmann-Deilmann GmbH, Germany) for 10 days in the greenhouse at 23°C daytime and 20°C night with a photoperiod of 16 hours. The primary leaves were then detached and transferred onto phyto agar plates containing 0.34 mM benzimidazol. *H*. *vulgare* cv. Ingrid wild type (WT) was used as a genotype susceptible to powdery mildew. The corresponding near-isogenic line Ingrid M.C. 20, containing dysfunction in mildew locus o 3 (*mlo3*) [26] was used to assess non race-specific papilla-based resistance. *H*. *vulgare* cv. Pallas 01, with resistant mildew locus a 1 and a 12 (*Mla1*; *Mla12*) was used to analyze a hypersensitive response [27, 28].

### Pathogen and inoculation

The barley *mlo3* and *Mla1* avirulent *Bgh* isolate K1 was used to analyze resistance reactions 0–120 hours after inoculation (hai) [29, 30]. *Bgh* K1 was maintained on cv. Tocada (KWS, Einbeck, Germany) in a controlled environment. Twenty-four hours before inoculation the conidia of heavily infested plants were shaken-off and discarded in order to assure homogenous and vital conidia for inoculation. Detached leaves on phyto agar were inoculated with a density of $\bar{x}$ = 329 (± 107) conidia/cm^2^ from young powdery mildew pustules (7–10 days after inoculation (dai) with *B*. *graminis* f. sp. *hordei*). The agar plates were sealed and incubated in a climate chamber at 19°C, 1100 m^−2^ *cd* illuminance and a photoperiod of 16 h per day. Development of *Bgh* and processes during resistance response of barley were histologically assessed, using Coomassie Brilliant Blue R-250 (CBB) and 3,3’-diaminobenzidine (DAB) staining [31, 32].

### Total RNA extraction, reverse transcription-polymerase chain reaction and real time qPCR

For each time point and treatment, RNA was isolated and purified in three independent experiments from five randomly pooled LN_2_ homogenized barley leaves (~1 g powdered material) using NucleoSpin RNA Plant kit and NucleoSpin RNA Clean-up kit (Macherey- Nagel, Düren, Germany), according to the manufacturers recommendations. Total RNA was determined by qPCR using GoTag G2 Hot Start Colorless Master Mix (Promega, Madison, USA). Therefore, the housekeeping genes *UBIQUITIN* and *Actin* were amplified, including genomic DNA as control sample. Subsequently, the qPCR product and RNA samples were stained with ethidium bromide in 2% agarose gel to detect contaminations with genomic DNA. Samples with DNA contamination were digested with DNase using TURBO DNA-free kit (Life Technologies, Carlsbad, USA) followed by the previous control step. The amount and quality of RNAs were photometrically analyzed using NanoDrop 2000 UV-VIS spectrophotometer (Thermo Scientific, Wilmington, USA).

For cDNA reverse synthesis 1 μg RNA was used. High-Capacity cDNA Reverse Transcription kit with RNase inhibitor (Applied Biosystems, Foster City, USA) was used for reverse transcription polymerase chain reaction following the manufacturer’s instructions. The cDNAs were checked by ethidium bromide staining in 2% agarose gel. Before performing real time qPCR, cDNA was diluted 1:50 with nuclease free water. PCR program was used for the Fast SYBR Green Master Mix (Applied Biosystems, Foster City, USA) with adjusted annealing temperature to the primers used. The amplified genes of interest were quantified in a relative way to the appropriate mock samples and the fold change in gene expression was calculated via the 2^−ΔΔCt^ -method by Livak and Schmittgen [33], using *UBIQUITIN* cDNA amplification for normalization. Used DNA oligonucleotide primers are shown in supplementary S1 Table. Amplified real-time PCR products were proved on 2% agarose gel after melting curve analysis at 55 − 95°C. Significant differences within the gene expression profile of the genes are analyzed by a Tukey-HSD Test with alpha = 0.5.

### Hyperspectral image acquisition, reflectance extraction and histological observation

Hyperspectral images were acquired with a hyperspectral line scanner (ImSpector PFD V10E, Spectral Imaging Ltd., Oulu, Finland) within a spectral range of 400 to 1000 nm and a spectral resolution of 2.73 nm, installed on a microscope [34]. Hyperspectral measurements were performed without ambient light. Samples were illuminated with two linear light emitters (Dual line Lightlines, Schott, Mainz, Germany) in a vertical orientation of 30° and a distance of 20 cm to the sample besides the fore optic. As a light source, a 150 watt halogen tungsten lamp connected to the line lights via a non-absorbing fiber was used (DCR Light Source EKE, Polytec, Waldbronn, Germany). Hyperspectral Spectral binning and spatial binning were set to 1. Frame rate and exposure time were adjusted to the object. A magnification of 7.3x (~7.5 μm per pixel spatial resolution) was used. Hyperspectral data cubes were assessed from four healthy and eight with powdery mildew inoculated leaves per genotype, every 3 hours from 0 hai until 48 hai, followed by daily measurements until 120 hai. To receive the relative reflectance a white reference bar (SphereOptics GmbH, Uhldingen-Mühlhofen, Germany) with a 100% reflection was recorded (W), followed by a dark current image (B_1_) to inherent the irradiance of the sensor. Subsequently, the leaf sample (I_0_) and a corresponding dark current image (B_2_) was recorded. Calculation of relative reflectance was according to the formula (I = (I_0_-B_2_)/(W-B_1_)), using the software ENVI 5.1 + IDL 8.3 (ITT Visual Information Solutions, Boulder, USA). Following, spectral signals were smoothed by employing the Savitzky-Golay filter [35] and cutting reflectance values below 420 nm and above 830 nm because of high measurement noise. Parameters for the Savitzky-Golay filter were 25 centered supporting points and a third degree polynomial.

Spectral signatures of pixels from healthy and diseased regions were extracted manually. Therefore, a region of interest of about ~1,000,000 pixels was extracted for every image of all screened leaves. The arithmetic average spectrum was calculated for the regions of interest as the basis for further analysis. Differences in hyperspectral reflectance among genotypes and over time were calculated according to Carter and Knapp [36]. Data mining approaches inspired by Kuska et al. [34], and Kuska et al. [37], were used to compute binary maps for *Bgh* disease detection on WT and *mlo3* barley as well as HRs on *Mla1*. Simplex Volume Maximization (SiVM) was applied to determine extreme signatures from hyperspectral images and all signatures were represented as a linear combination of these selected extremes. Thereby, these extremes are real spectral signatures, that are interpretable and uncover the variation existing in the data. In addition, the new representation enables expression of specific signatures as level of transition between healthy and diseased leaves [11, 34, 38] and the algorithm scales well with data dimensionality and size enabling statistical data mining on a massive scale e.g. in high-throughput hyperspectral imaging of plants [39]. In these experiments, the number of extremes computed by SiVM were set to *k* = 25. Differences in probability based on the simplex distributions of healthy and inoculated samples were then used for highlighting potentially affected regions.

### Linear Discriminant Analysis for the visualization of synergetic effects

Linear Discriminant Analysis (LDA) can reduce the dimensionality of a dataset retaining the separability of the classes within the dataset. Visualizing the low-dimensional representation reveals the suitability of the features to separate the classes. This allows classification of an unknown sample as a member of a particular group. As an example, spectral investigation with different fruits applied successfully the LDA to distinguish fruits and vegetables based on the spectral properties of the parenchymal cell walls [40]. In addition due to its low model complexity, LDA is common in bio-statistical approaches with many generalizations and regularizations e.g. in microarray analysis [41].

LDA included in Matlab 2013a (The MathWorks Inc., Massachusetts, USA), was applied to three different views on the plant status: (I) spectral reflectance, (II) gene activity and (III) the combination of both. For the calculation of the LDA transformations to two dimensions, each spectral observation is represented by five evenly distributed bands and each gene activity measurement is represented by three technical repetitions. The combined feature set includes all features using feature stacking. Direction of the vectors was calculated by changed center of gravity during the experimental period.

### RELIEF algorithm for feature selection

**Fig 1:**
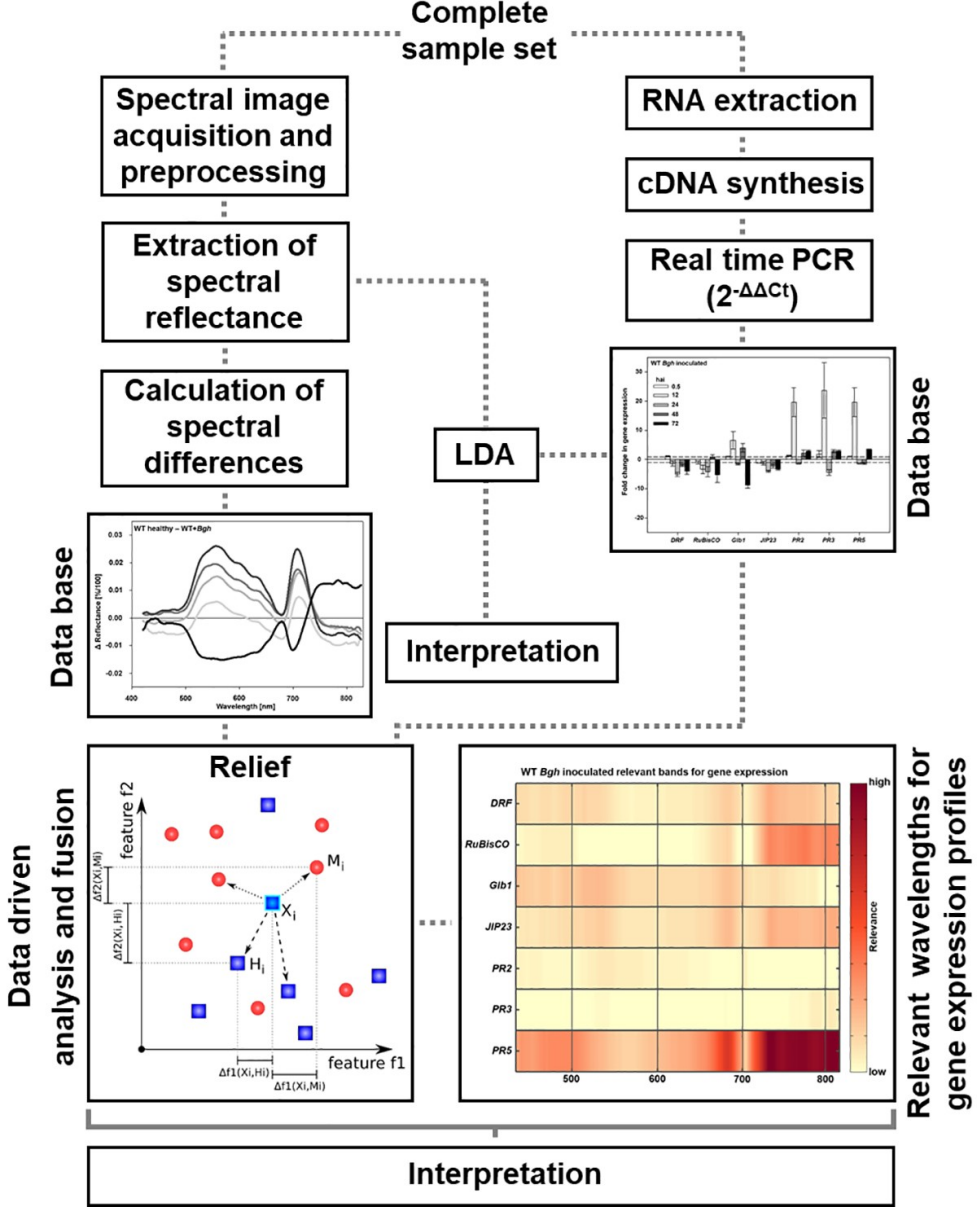
Linking hyperspectral imaging data with gene expression profiles. Healthy and *B*. *graminis* f.sp. *hordei* inoculated primary leaves were used for hyperspectral imaging and RNA extraction. After hyperspectral image acquisition and normalization, the Savitzky-Golay-filter was applied to reduce measurement noise. Reflectance values were manually extracted from regions of interest and used to identify relevant time points in the barley-*Bgh* interactions by applying the Relief algorithm. Spectral reflectance of identified relevant time points were used to calculate spectral differences between healthy and *Bgh* inoculated barley. In parallel, RNA was used for reverse transcriptase to synthesize cDNA and to analyze gene expressions of selected genes using real time PCR. Linear Discriminant Analysis (LDA) was applied on the hyperspectral data, gene expression profiles and a combination of both data sets to visualize the suitability of the features for the separation of different barley-*Bgh* interactions. To determine relevance as indicator for interpretable relations between hyperspectral signatures and gene expression profiles the Relief algorithm was applied (blue = high relevant wavelength band).

The selection of relevant features is an important preprocessing step for the reduction of evaluation time and improvement of result quality in hyperspectral data analysis. For the assessment of feature relevance, multiple methods were developed [42]. Within this study the regressional RELIEF-F (Relief) algorithm for feature selection included in Matlab 2013a (The MathWorks Inc., Massachusetts, USA) was used to gather additional information about the relationship between single spectral bands and plant-physiological properties (Fig 1). The Relief algorithm as a filter algorithm, determines the relevance of features independently from a specific prediction algorithm. The algorithm is based on the idea that the values of relevant features are not arbitrarily distributed in the feature space but that they are grouped by corresponding classes [43]. By analyzing the local neighborhood for all data points of a data set, the local characteristics can be transferred to the suitability of a single feature for a specific prediction tasks [44]. In the classification case (Fig 1), the difference Δ in a feature value f_j_ of a data point *X*_*i*_ to the next data point from its class *H*_*i*_ (hit) and from a different class *M*_*i*_ (miss) is transferred to the relevance R_*i*_(f_*j*_) = Δ*f*_*j*_(*X*_*i*_,*M*_*i*_)−Δ*f*_*j*_(*X*_*i*_,*H*_*i*_). Averaging the relevance across all *n* data points *X*_*i*_, *i = 1…n* gives a global estimation of feature relevance based on the local characteristics.

In this work, the Relief algorithm was used to determine the relevance as an indicator for interpretable relations within the data. It was applied to investigate the relevance of single wavebands of each genotype after inoculation. The significance of these differences were tested by applying the Welch's t-test (Supplementary S1 Fig). In addition, it was also possible to evaluate differences in the relevance of gene expression activities for the hyperspectral reflectance characteristics. This method has been used to select informative genes for cancer classification using microarray gene expression data and for the development of spectral indices for plant disease detection [45, 46].

## Results

### Relevance of spectral wavelengths for discrimination of non-inoculated and inoculated barley

**Fig 2:**
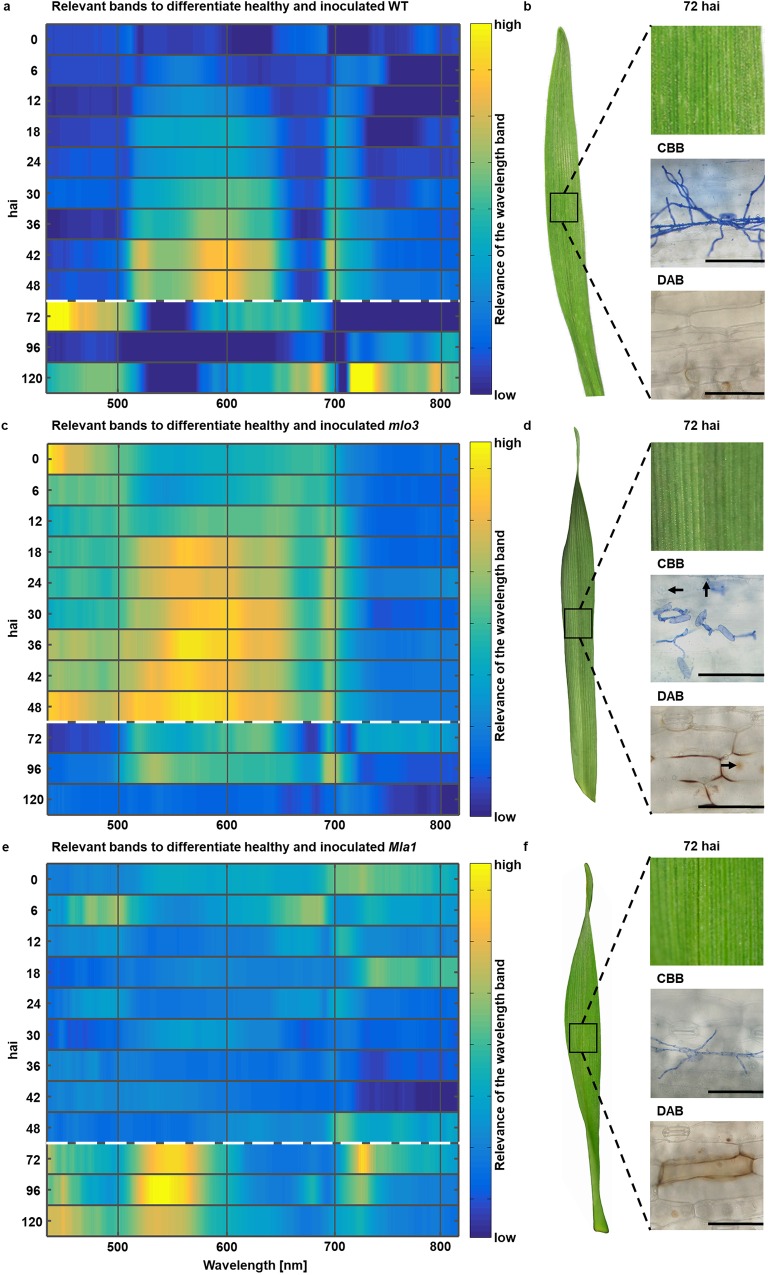
Identifying relevant time points during different barley-powdery mildew interactions. **a,c,e,** Relevant wavelength bands during susceptible WT as well as resistant *mlo3* and *Mla1* barley-*B*. *graminis* f.sp. *hordei* (*Bgh*) interactions 0–120 hours after inoculation (hai). Dashed lines indicate changed measurement frequency in the experimental period. **b,d,f,** Corresponding RGB images were determined to analyze the phenotype 0, 24, 48 and 72 hai. Histological observations using Coomassie Brilliant Blue R-250 (CBB) and 3,3’- diaminobenzidine (DAB) were used to assess the development of *Bgh* and resistance responses of barley. Arrows indicate formed papilla in *mlo3* leaves (n = 8 replicates. Scale bars correspond to 100 μm).

To discriminate between healthy and *Bgh* inoculated leaves, relevant wavebands were determined using the Relief algorithm (Fig 2A, 2C and 2E). Therefore, the hyperspectral reflectance of healthy and *Bgh* inoculated leaves were used as one data set in the Relief algorithm. The relevance of a waveband, to distinguish between healthy and *Bgh* inoculated, is indicated from blue (low relevance) to yellow (high relevance).

Hyperspectral reflectance from 0 to 120 hai were analyzed to identify key time points of changes in spectral reflectance for the different interactions. The compatible interaction (WT) reveals relevant wavelength from 520–660 nm and around 690 nm, at 6 hai and 48 hai (Fig 2A). Waveband range of 400–520 nm shows high relevance 72 hai and 120 hai. The near infrared (NIR, 700–830 nm) range indicates higher relevance 36–48 hai and 96–120 hai, especially around 720 nm 120 hai. Secondary mycelia have grown at 72 hai on the WT, visualized by CBB staining (Fig 2B) and no significant H_2_O_2_ generation could be observed 72 hai. Visible powdery mildew symptoms appeared 120 hai (not shown).

*Bgh* inoculated *mlo3* leaves showed high spectral relevance in wavelengths from 400–720 nm 0–48 hai (Fig 2C). Wavebands with the highest relevance to discriminate between healthy and inoculated *mlo3* leaves were around 570 nm 36 hai and 48 hai. Later time points show similar relevance over the entire spectrum. The leaves did not show any symptoms. Microscopic observations at 72 hai by CBB and DAB staining indicated fully developed papilla, which stopped *Bgh* penetration and fungal development (Fig 2D). Wavelengths with high relevance to determine inoculated and to distinguish healthy *Mla1* leaves, were indicated by the Relief algorithm at 400–600 nm and around 730 nm 72–120 hai (Fig 2E). Wavelengths in the blue range provide relevance 12 and 24 hai. H_2_O_2_ generations were microscopically confirmed 72 hai using DAB staining, which indicates HRs against *Bgh* (Fig 2F). After *Bgh* penetration, pathogen development was stopped by HR at *Mla1* genotypes (Fig 2F, CBB).

Overall, the Relief algorithm identified relevant changes in the spectral reflectance during early plant-pathogen interaction for all three barley-powdery mildew interactions specifically 12, 24, 48, 72 and 120 hai. The significance of spectral changes at these time points was analyzed using the Welch’s t-test (Supplementary S1 Fig). These specific time points were further used to calculate differences in the hyperspectral reflectance between healthy and *Bgh* inoculated leaves to assess subtle changes during barley-*Bgh* interactions.

### Early hyperspectral reflectance differences between susceptible, *mlo* and *Mla* resistant barley during interaction with *Bgh*

**Fig 3:**
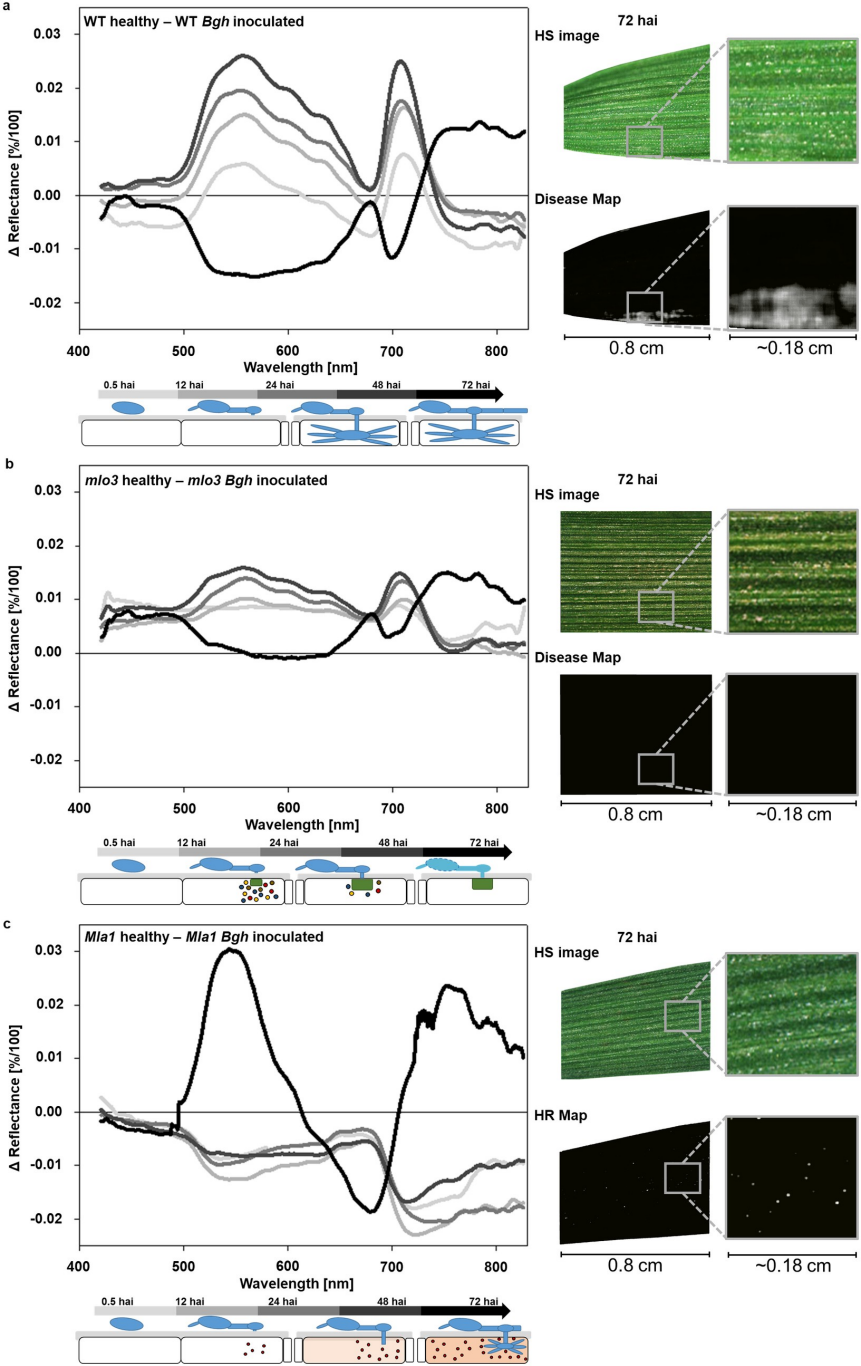
Assess spectral reflectance signatures by difference calculation. **a,b,c** Spectral differences of healthy and inoculated with *B*. *graminis* f.sp. *hordei* (*Bgh*), susceptible WT, resistant *mlo3* and *Mla1*, 0.5, 12, 24, 48 and 72 hai. Positive values in the difference plot demonstrate higher reflectance intensity of healthy leaves, negative values higher reflectance intensity of inoculated leaves. In addition, schemes of the interaction types development are illustrated. On pseudo RGB image from HSI, no symptoms are visible. Based on the reflectance spectrum, computed disease maps indicate *Bgh* infested pixels in white 72 hai. HR maps indicated pixels, which undergoes a hypersensitive response also in white 72 hai. (n = 8 biological replicates).

To identify differences in the spectral pattern during barley-*Bgh* interactions, the difference of hyperspectral reflectance intensity between healthy and *Bgh* inoculated leaves were calculated (Fig 3). Spectral differences were calculated for 0.5, 12, 24, 48 and 72 hai. At these time points symptoms were not macroscopically visible (Fig 3). First symptoms occurred 120 hai for the naked eye (not shown). For this reason, this time point was used as a macroscopically positive control but was no longer considered for further analysis. Instead, spectral signatures at 0.5 hai were used to assess influence of *Bgh* inoculation on the spectral reflectance. Calculations of difference spectra allowed an explicit analysis of significant wavelength ranges between healthy and inoculated genotypes (Fig 3). All minus values correspond to a higher reflectance intensity of healthy leaves and all plus values to a higher reflectance intensity of *Bgh* inoculated leaves. Differences between healthy and inoculated WT leaves were marginal around ~0.0024 [Δ%/100] mainly in the NIR range 0.5 hai (Fig 3A). The reflectance intensity of healthy WT leaves increased 400–670 nm and around 710 nm 12–48 hai. During these points in time, reflectance in the NIR range was slightly higher in WT inoculated leaves. Conidia already penetrated epidermal cells and haustoria were developed. The spectral pattern changed by an increased reflectance at 490–670 nm and around 700 nm and a decreased reflectance in the NIR 72 hai, investigated by the calculated difference signature (Fig 3A). At this timepoint, *Bgh* is in the post penetration phase including first full developed haustoria and secondary mycelia. Computed disease maps indicate powdery mildew infested spots 72 hai.

Spectral differences between healthy and inoculated *mlo3* leaves exhibit a different pattern (Fig 3B). Reflectance intensities were higher on healthy leaves over the entire spectrum 0 to 48 hai. During this time span, effective papillae developed in *mlo3* inoculated leaves, but only marginal changes in the spectrum could be observed. Highly significant spectral changes were shown 400–720 nm 72 hai (Fig 3B and Supplementary S1B Fig). The disease map of inoculated *mlo3* shows only black pixels, indicating healthy tissue.

*Bgh* inoculated *Mla1* show higher reflectance intensities at 500 to 850 nm from 0.5 until 48 hai (Fig 3C). Early differences in reflectance were found in the NIR especially around 710 nm and 780 nm with a difference up to 0.027 [Δ%/100] 12 hai. During this time point of interaction, *Bgh* has developed an appressorium and starts to penetrate the epidermal cells and subsequently on the *Mla1* genotype a HR is induced. During the HR process, spectral difference pattern changed 72 hai (Fig 3D). These spectral changes were highly significant from 500–650 nm and 690–830 nm (Supplementary S1C Fig). HR spots were not visible for the naked eye until 72 hai. Nevertheless, the applied HR map indicated pixels undergoing a HR in white.

### Gene expression profiles during barley-powdery mildew interactions

**Fig 4:**
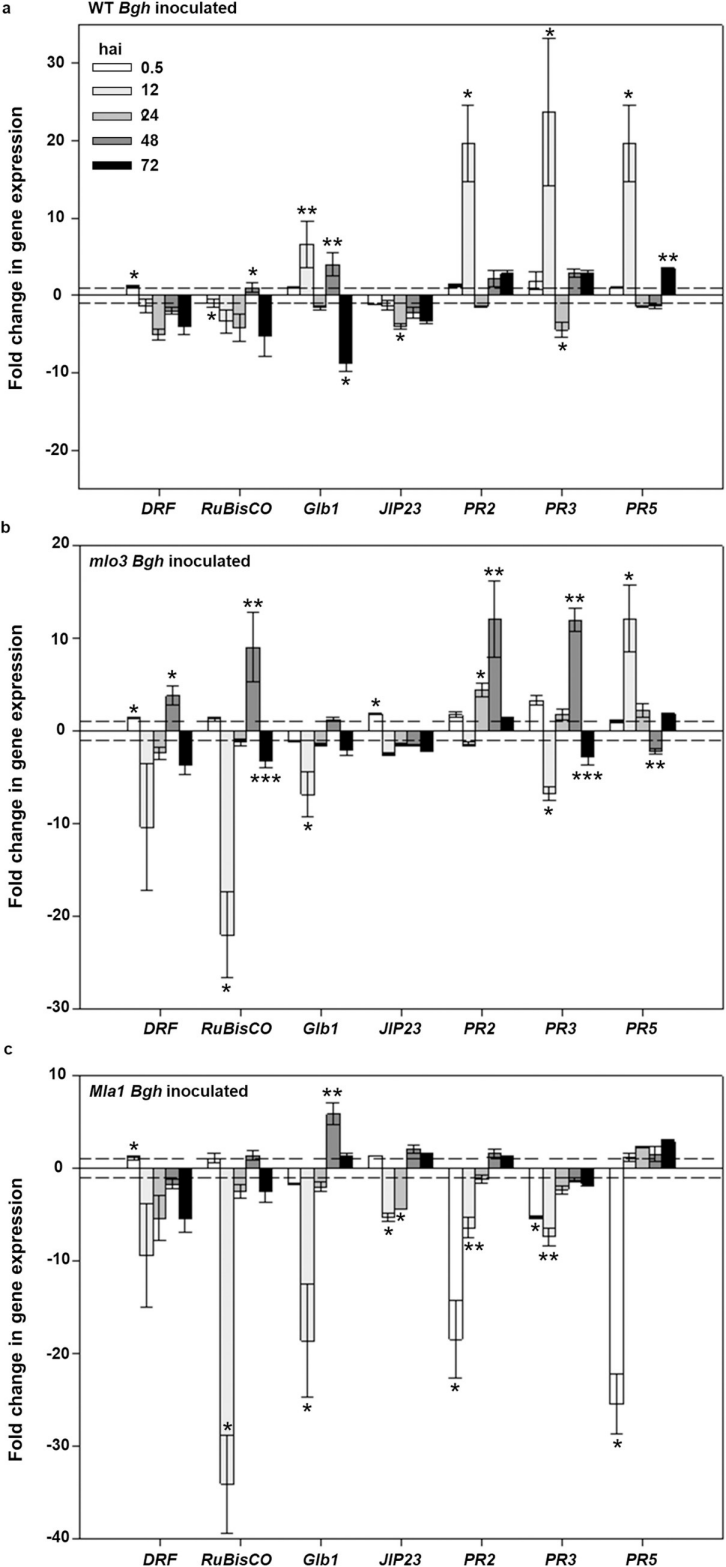
Gene expression profiles of seven genes affected during different barley-powdery mildew interactions. **a,b,c,** Gene expression profiles of inoculated with *B*. *graminis* f.sp. *hordei* (*Bgh*), susceptible WT, resistant *mlo3* and *Mla1*, normalized to their appropriated mock samples 0.5, 12, 24, 48 and 72 hai. (n = 3 replicates; error bars indicate standard deviation; asterisks show significant differences within the gene expression profile per gene calculated with Tukey-HSD, α = 0.05).

In order to elucidate transcriptional activity of near isogenic barley lines during *Bgh* interaction, seven genes related to senescence and cell metabolism, cell signaling and resistance response were analyzed 0.5, 12, 24, 48 and 72 hai. *Bgh* inoculated WT showed down-regulated senescence corresponding genes indicated by a negative change of *HvDRF* and *HvRuBisCO* (Fig 4A). *HvDRF* was down-regulated from 24 until 72 hai. In contrast, *HvRuBisCO* was down-regulated already 12 until 72 hai without significant differences compared to healthy leaves 48 hai. Resistance responses by WT leaves against *Bgh* were indicated by up-regulated *PR* genes 12 and 72 hai. Differences in transcriptional activity between *HvPR2*, *HvPR3* and *HvPR5* during 24 and 48 hai were present. Interestingly, *HvGlb1* showed higher transcriptional activity 12 and 48 hai in inoculated WT leaves according to *Bgh* penetration time points. Down regulation of *HvJIP23* from 24 hai suggest that jasmonic acid is not an early key factor in this interaction system.

Gene expression-profile for *mlo3* inoculated leaves exhibit a different pattern compared to the other genotypes and treatments (Fig 4B). *HvPR3* codes a chitinase 2 and was already upregulated 0.5 hai. All tested genes were then down-regulated 12 hai, except *HvPR2* which had a higher transcriptional activity until 48 hai. *HvDRF*, *HvRuBisCO* and *PR* genes had a higher transcriptional activity in inoculated leaves compared to healthy *mlo3* leaves 48 hai.

Gene expression-profile of *Mla1* inoculated leaves refer to an initiate cellular degradation approved by down regulated gene expression of mostly all genes 12 hai (Fig 4C). *HvGlb1* and *HvJIP23* were the only genes in this system, which were significantly up regulated 48 hai.

### Interrelations between hyperspectral signatures and gene expression profiles

**Fig 5:**
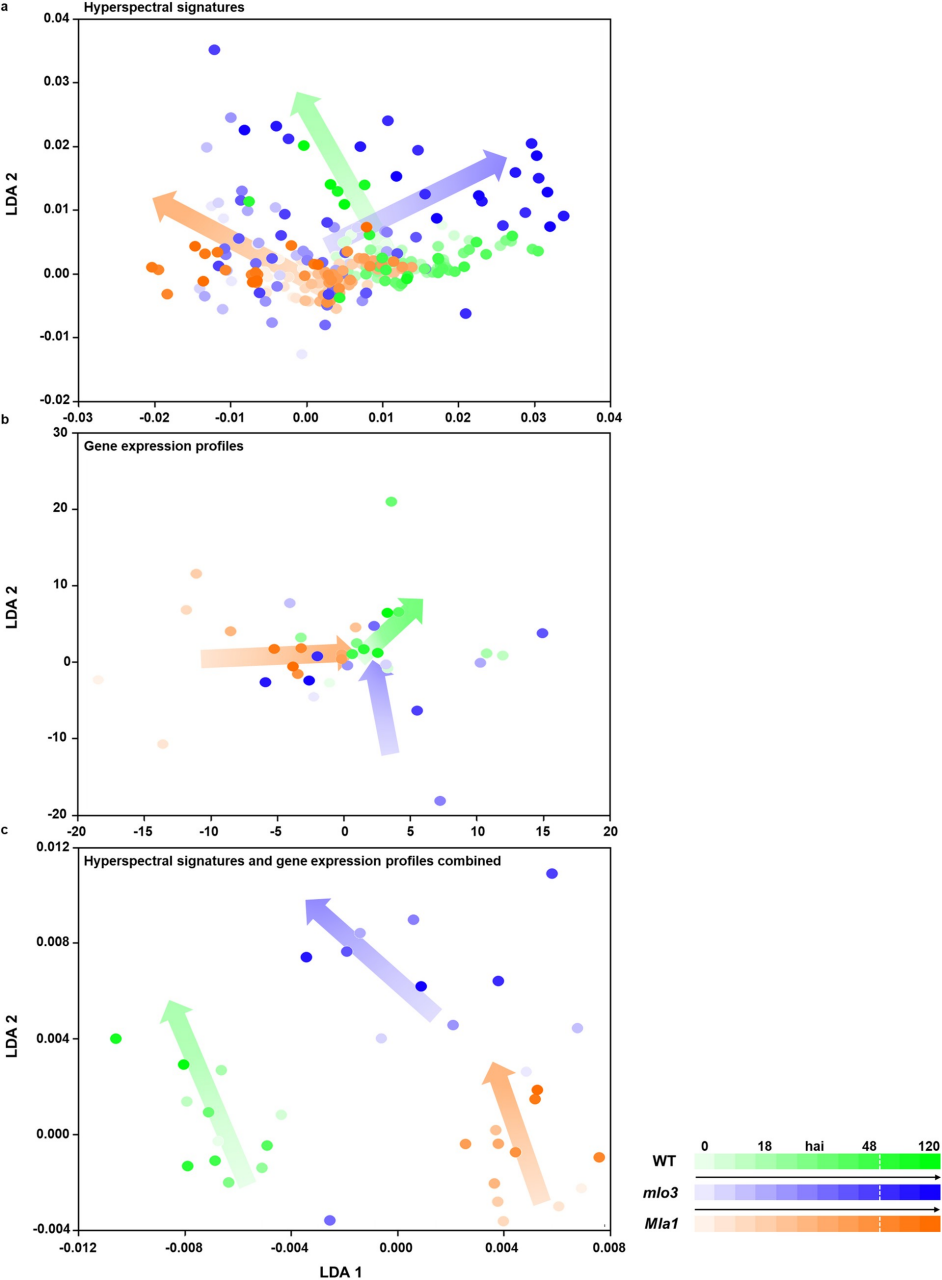
Feature detection by Linear Discriminant Analysis (LDA). **a,b** The suitability of features to differentiate *Bgh* inoculated susceptible wild type (WT) barley, papilla resistant (*mlo3*) and hypersensitive resistant (*Mla1*) barley were tested using a LDA on hyperspectral signatures, gene expression profiles and the combined data sets 0–72 hai. **c**, Combining both data sets increased the separation between the different interaction types WT, *mlo3* and *Mla1*. (Arrows indicate the changed centre of gravity per barley-*Bgh* interaction over the experimental period).

To discriminate early *Bgh* pathogenesis on WT leaves and resistance responses on *mlo3* and *Mla1* leaves, LDA was applied on hyperspectral signatures, gene expression profiles and combined data sets (Fig 5). The LDA classification by hyperspectral signatures revealed an improved discrimination of the barley-*Bgh* interaction types with ongoing pathogenesis (Fig 5A). In contrast, LDA classification of the gene expression profiles of *Bgh* inoculated barley genotypes carried out a discrimination during the early time points (Fig 5B). Applying the LDA on the combined data sets of hyperspectral signatures and gene expression profiles revealed an efficient discrimination between the susceptible WT, *mlo3* and *Mla1* based resistant barley (Fig 5C). This shows significant synergistic effects between the hyperspectral signal and the corresponding gene activities carving out the subtle differences between the early plant adaptions.

### Relevant wavelength bands for the individual gene expression profiles

**Fig 6:**
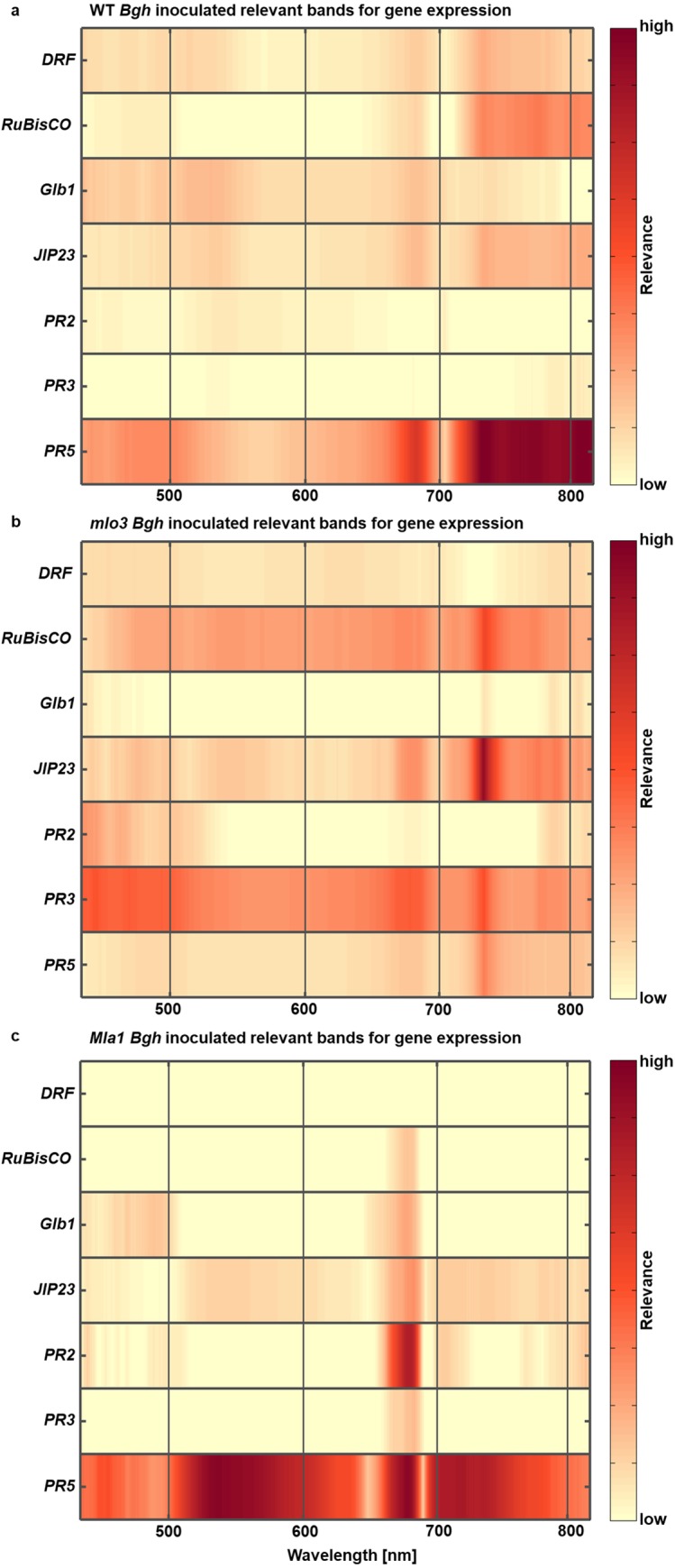
Relief algorithm to assess differences in the relevance of individual genes per wavelength band. **a,b,c** High relevant wavelength bands for specific gene expression profiles of barley genotypes inoculated with *B*. *graminis* f.sp. *hordei* (*Bgh*) are indicated in dark red and low relevant wavelength bands in beige.

To analyze the coherence between spectral reflectance and gene expression profiles during barley-*Bgh* interactions, relevant wavelength bands and reflectance intensities for each gene were computed using the Relief algorithm. Wavebands with high relevance are indicated in dark red, low relevance is indicated by beige color (Fig 6).

Relevant wavebands for *HvDRF* expression profile of *Bgh* inoculated WT are around 730 nm (Fig 6A). The NIR range is relevant for the expression profile of *HvRuBisCO*, *HvJIP23* and *HvPR5*. Wavebands at 400–530 nm and around 680 nm are relevant for *HvPR5* expression during WT-*Bgh* interaction, respectively.

Expression profile of *HvRuBisCO* of *Bgh* inoculated *mlo3* had varying relevant wavebands in the VIS-NIR range with the highest relevance around 730 nm (Fig 6B). High relevance around 730 nm and 760–830 nm are shown as well for *HvJIP23*. *HvPR3* expression profile was related to reflectance at 400–530 nm, around 690 nm and 730 nm.

Relevant wavebands for gene expression profiles of *Bgh* inoculated *Mla1* leaves were mainly computed from 660–680 nm for almost all genes. Thereby, *HvDRF* profile shows no relevant wavebands in the VIS-NIR range. Wavebands from 520–650 nm, 660–680 nm and 700–750 nm show high relevance for the expression profile of *HvPR5*.

## Discussion

### *Bgh* influence early spectral characteristics of barley

Previous studies on hyperspectral imaging of barley-*Bgh* interactions elucidated spectral characteristics of powdery mildew pathogenesis, as well as for *Mla1* and *mlo3* based resistance between 0 until 14 dai [34, 38]. Important time points varied depending on the interaction. Investigations by Kuska et al. [37] indicated key moments in the plant-pathogen interaction to distinguish barley-*Bgh* interactions by hyperspectral imaging and histologic analysis 2–3 dai. In the present study the relevance of spectral changes during barley-*Bgh* interactions were revealed for the first time. Hyperspectral imaging of early time points (0–48 hai) revealed changes in the metabolism of inoculated WT and *mlo3* near-isogenic lines. During this interaction time, senescence was decelerated in inoculated leaves, indicated by stable reflectance intensity in the green range.

Barley leaves with a dysfunctional *mlo* gene had fully developed papillae 24–48 hai as described by Thordal-Christensen et al. [32] and Hückelhoven et al. [14]. The former, complex cell wall aggregation [47] results in decreased reflectance from 420–660 nm and around 710 nm 1–2 dai. This may be explained by cytoplasmic streams in attacked epidermal cells [48]. Similar spectral pattern for *Bgh* inoculated *mlo3* leaves were shown by Kuska et al. [37], already 2 dai. *Mla1* based hypersensitive reaction, which cause an early change 12 hai in the NIR range, is correlated with plant physiological and structural changes [37]. This indicates a rapid mode of action within the first 24 hours according to described process of *Mla* gene based HR [14]. This process seems to decelerate 72 hai, displayed by a turnover of the difference reflectance. In previous studies, this turnover was spectrally determined in a *Bgh* inoculated *Mla1* already 48 hai [37]. This indicates a temporal dynamic of the *Mla* response against *Bgh* penetration. Nevertheless, the spectral difference pattern is similar after the HR undergoing cells are necrotized.

### Gene expression profiles and their link to hyperspectral reflectance

The LDA analysis on hyperspectral signals or gene expression profiles indicated a high dispersion. This phenomenon derived from the temporal variability during the early individual interactions that depend on the *Bgh* development and penetration time point [37, 38]. Differences in the separability result from complex physiological and biochemical processes triggering the gene to phenotypic function and are based on the corresponding analysis methods [49]. In contrast, applying the LDA on the combined data sets differentiate all three barley-*Bgh* interactions independent from the time. This shows significant synergistic effects between the hyperspectral signal and the corresponding gene activities. These results lead to demand for in depth analysis on the temporal variability of the hyperspectral signals and temporarily corresponding gene expression profiles, to elucidate a possible functional link between both highly diverse data sets.

Depending on the barley-*Bgh* interaction, different genes were expressed. The *Bgh* infestation begins with the conidia developing an appressorium and starting to penetrate the epidermal cell wall using cell wall degrading enzymes and by an increased turgor pressure [50]. At this time point, *PR* genes are induced and a down regulation of *HvRuBisCO* has been described in studies by Eichmann et al. [23]. During the compatible interaction, *PR5* showed broad functionality indicated in high frequency of relevant wavebands. *PR5* is reported as a marker for systemic acquired resistance, but transcribed proteins are endo-*ß*-1,3-glucanases as well as endo-*ß*-1,4-xylanases with anti-fungal activity [19]. A possible xylanase activity is indicated by high relevant wavelengths around 710 nm. Reflectance intensity around 700 nm is characterized by leaf and cell structure [51]. The cell structure is highly influenced by the cell wall in which xylan is a major component for the cell wall of monocotyledonous plants and can be degraded by endo-*ß*-1,4-xylanases [52]. This coherency indicates that *PR5* activity is important during compatible barley-*Bgh* interaction.

Further important gene activity during compatible barley-*Bgh* interaction was revealed by upregulated *HvGlb1*, which is stimulated by gibberellic acid and transcripts of *ß*-1,3-glucanase mainly in aleuron cells to hydrolyze the cell walls [53]. Previous studies indicate an early up-regulation of *ß*-1,3-glucanases in *Bgh* inoculated barley leaves [54]. Similar upregulation profiles are caused by necrotrophic plant pathogens such as *Fusarium graminearum* infestation on barley roots, which underlines a basal resistance response [55]. Interestingly, a second overexpression of *HvGlb1* was during secondary mycelia growth, which initiates a second penetration phase. The highest relevant wavelength bands for *HvGlb1* expression profile are in accordance with the mainly influenced wavelengths during early powdery mildew pathogenesis, which reveals *HvGlb1* as a possible *Bgh* penetration indicator [9, 34, 37].

The influence of *Bgh* on expression of *JIP23* was as expected. After *Bgh* invaginates the epidermal cell and develops a functional haustorium, effector proteins are secreted by *Bgh* to successfully infest the plant and to overcome plant immunity [56]. Investigation by Scheler et al. [57] provided evidence of early *HvJIP23* transcript suppression due to *Bgh* infestation, which is strongly linked to jasmonate activity and its associated induced systemic resistance [21]. In addition, jasmonates cause loss of chlorophyll, underlining their role in leaf senescence [20]. Relevant wavelength bands around 550 nm, 640–690 nm and 720–830 nm for *HvJIP23* expression profiles in *Bgh* inoculated WT leaves are known to correlate to the chlorophyll content, the photosynthetic activity and the leaf cell structure [51, 58].

### HSI reveals the metabolism during incompatible barley - powdery mildew interactions

Upregulated *HvDRF* and *HvRuBisCO* in *Bgh* inoculated *mlo3* are evidence for a finished resistance response [59]. This result is in accordance with studies by Swarbrick et al. [22] that analyzed a high transcript amount of *RuBisCO* related genes in a *Bgh* inoculated *mlo5* genotype from 3 dai. The *HvRuBisCO* expression profile in this study underlines relevant wavebands in the green and red range, elucidating the coherency of *RuBisCO* to the photosynthesis apparatus [60, 61]. In addition, wavelength bands in the NIR are relevant due to the influence of *RuBisCO* on barley leaf cell development [62].

Further spectral coherency of gene expressions in *mlo3* were revealed for *HvPR3* expression, which is not known to be directly involved in barley *mlo* base resistance response. Relevant wavelength bands for *HvGlb1* expression profiles in *Bgh* inoculated *Mla1* reveals important spectral ranges for early *Bgh* infestation. If *HvGlb1* is a potential *Bgh* penetration indicator, these results are in accordance with studies by Kuska et al. [37]. They observed several successful penetrations of *Bgh* on resistant *Mla1* leaves that were later stopped by strong HRs. *HvPR2* and *HvPR5* expression profiles and corresponding relevant wavelengths underline the important role of *HvPR* gene regulation during HR and cell collapsing against powdery mildew infestation [63].

The link between phenomic data and known parameters from omic and physiological studies is highly demanded to establish high-throughput phenotyping pipelines in crop breeding and crop improvement [4, 64]. Results of the present study successfully demonstrate a link between gene expression and hyperspectral reflectance. According to the hypotheses by Furbank and Tester [2], it is also possible to study the function of genes using the phenomic information derived from the hyperspectral reflectance. The Relief algorithm determined the relevant wavelength bands for a specific gene expression profile and enabled the interpretation of the coherency based on the gene transcript responsibility. Until now, only a few studies have attempted to find these functional relations among spectral phenomics and genomic data. Recently, sugar beet inbred lines differing in two quantitative trait loci against *Cercospora* leaf spot could be differentiated according to disease severity using HSI [11]. Investigations by Kuska et al., [65] revealed that the combination of invertase analysis and multispectral imaging was a complementary validation system in a high-throughput approach. This provides evidence that spectral technologies bear the potential to be implemented in exhaustive breeding processes, but therefore all specific crop-pathogen interactions must be determined to assess the characteristic spectral properties. In a next step, the impact of proteins, secondary metabolites and hormones on spectral profiles during barley-*Bgh* interactions need to be investigated. This would reveal a comprehensive phenotyping of a genotype and will support the development of hyperspectral gene maps and improve existing crop physiological modelling e.g. [49]. Then, assessed relevant wavebands of specific gene/protein/metabolite activities need to be validated using knock-out mutants. This will establish novel approaches for plant-pathogen interaction studies and crop resistance breeding.

## Supporting information

S1 Fig Welch’s t-test to determine the significance of changes in hyperspectral reflectance. Significance is determined 0.5 to 12, 12 to 24, 24 to 48 and 48 to 72 hai of *B*. *graminis* f.sp. *hordei* inoculated susceptible wild type (WT), *mlo3* and *Mla1* resistance barley. High significance is indicated in white and low significance of the wavelength band is indicated in black.
(DOCX)

S1 Table Oligonucleotide primers for qPCR. The acquisition temperatures and PCR product sizes in base pairs for RT-qPCR synthesized cDNA for real time qPCR used in this study.
(DOCX)
