# Supplementary material for: Discovering coherency of specific gene expression and optical reflectance properties of barley genotypes differing for resistance reactions against powdery mildew
Source: PLoS One. 2019 Mar 19;14(3):e0213291. doi: 10.1371/journal.pone.0213291 (PMC6424429; doi:10.1371/journal.pone.0213291)
Supplement: S1 Fig — Significance is determined 0.5 to 12, 12 to 24, 24 to 48 and 48 to 72 hai of B. graminis f.sp. hordei inoculated susceptible wild type (WT), mlo3 and Mla1 resistance barley. High significance is indicated in white and low significance of the wavelength band is indicated in black. (DOCX) [file pone.0213291.s001.docx]

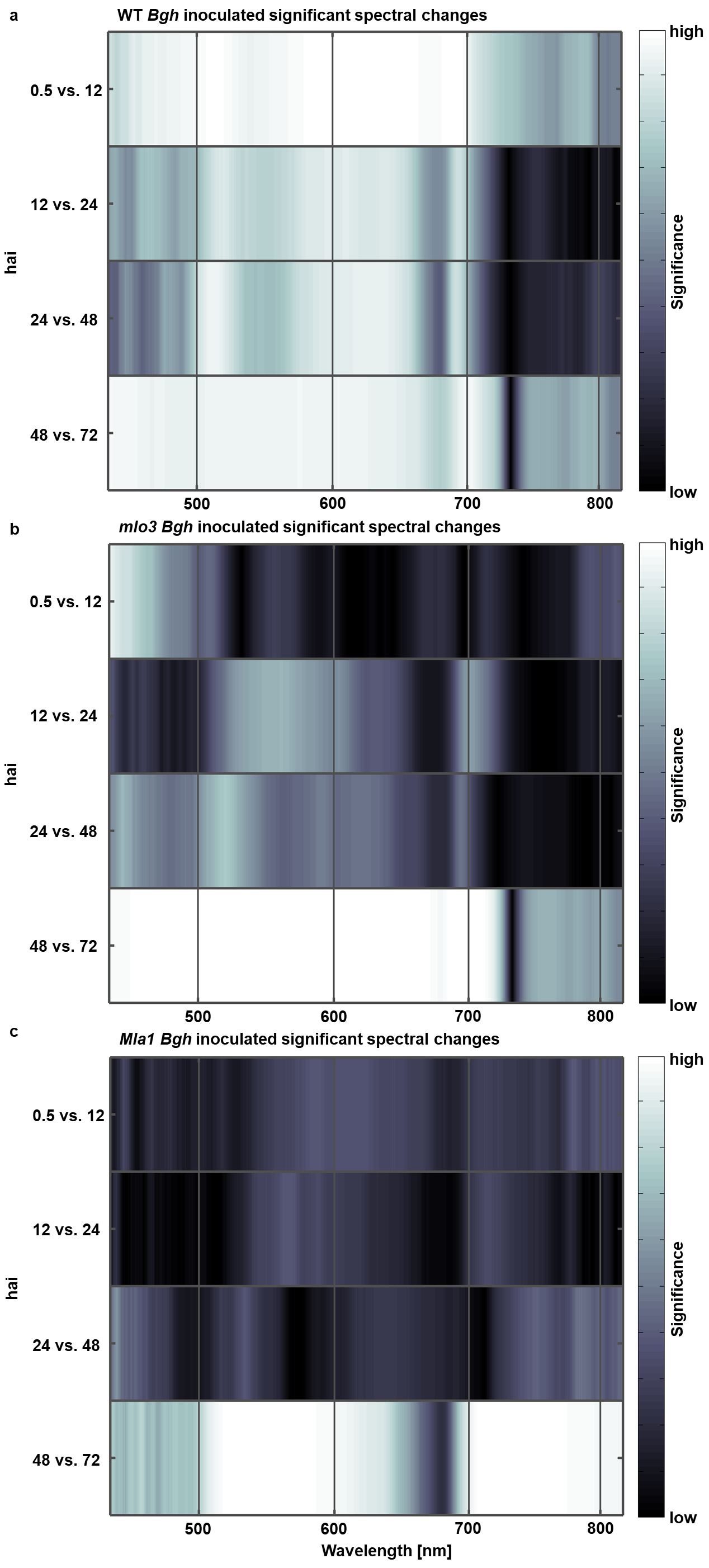


**Figure S1.** Welch’s t-test to determine the significance of changes in hyperspectral reflectance 0.5 to 12, 12 to 24, 24 to 48 and 48 to 72 hai of *B. graminis* f.sp. *hordei* inoculated susceptible wild type (WT), *mlo3* and *Mla1* resistance barley. High significance is indicated in white and low significance of the wavelength band is indicated in black.
