# Supplementary material for: Discovering coherency of specific gene expression and optical reflectance properties of barley genotypes differing for resistance reactions against powdery mildew
Source: PLoS One. 2019 Mar 19;14(3):e0213291. doi: 10.1371/journal.pone.0213291 (PMC6424429; doi:10.1371/journal.pone.0213291)
Supplement: S1 Table — The acquisition temperatures and PCR product sizes in base pairs for RT-qPCR synthesized cDNA for real time qPCR used in this study. (DOCX) [file pone.0213291.s002.docx]

**Table S1.** Oligonucleotide primers, acquisition temperatures and PCR product sizes in base pairs for RT-qPCR synthesized cDNA used in real time qPCR.

| Gene | Acession number | Forward primer (5´->3´)/reverse primer (5´->3´) | Annealing temperature (°C) | Product size (bp) |
| --- | --- | --- | --- | --- |
| *^1^UBIQUITIN* | M60175 | ACCCTCGCCGACTACAACAT / CAGTAGTGGCGGTCGAAGTG | 60 | 263 |
| *^2^HvActin* | AY145451 | GGAAATGGCTGACGGTGAGGAC / GGCGACCAACTATGCTAGGGAAAAC | 57 | 105 |
| *^3^HvDRF1* | AY223807 | TCCTCTCGGTCAGATTTGCTGG / ACAGTCACCGGGTCAACTTCC | 57 | 227 |
| *^4^HvRuBisCO* | U43493 | TCCTTTCCAGGGGCTCA / GAGGCAAGGCACCCACT | 57 | 223 |
| *^5^HvPR2* | Barley1_01637 | TACTTCGCGTACCGTGACAA / GTGTAGGTCAGCCCGTTGTT | 60 | 101 |
| *^6^HvPR3* | AJ276226 | CATCACGCAATCGGTGTTACG / TAGTCTCGTGGGAGGTCTGG | 57 | 192 |
| *^7^HvPR5* | AY839295 | GCCGACCAACTACTCAATGT / AGGGCAGGTGAAGGTGCT | 57 | 118 |
| *^8^HvJIP23* | X98124 | GGAGTGTTTGGTACCCCCAT / GGCACCAGTGGCATTGTAGA | 60 | 250 |
| *^9^HvGlb1* | X56775 | CACATCAAGGTGACCACGTC / GGGTAGATGTTGGCCATGAG | 60 | 200 |

**References for primer sequences**

^1, 4^Eichmann R, Bischof M, Weis C, Shaw J, Lacomme C, Schweizer P, Duchkov D, Hensel G, Kumlehn J, Hückelhoven R: BAX INHIBITOR-1 is required for full susceptibility of barley to powdery mildew. Molecular Plant-Microbe Interactions. 2010; 23:9:1217-1227.

2Janack B, Sosoi P, Krupinska K, Humbeck K. Knockdown of WHIRLY1 affects drought stress-induced leaf senescence and histon modifications of the senescence-associated gene HvS40. Plants. 2016; 5:37.

^3, 6^Primersequences designed according to Untergasse A, Nijveen H, Rao X, Bisseling T, Geurts R, Leunissen JAM. Primer3Plus, an enhanced web interface to Primer3. Nucleic Acids Research. 2007; 35: 71-74.

^5, 7^Molitor A, Zajic D, Voll LM, Pons-Kühnemann J, Samans B, Kogel KH, Waller F. Barley leaf transcriptome and metabolite analysis reveals new aspects of compatibility and *Piriformospora* *indica*–mediated systemic induced resistance to powdery mildew. Molecular Plant-Microbe Interactions. 2011; 24:1427-1439.

^8^Schäfer P, Pfiffi S, Voll LM, Chandler PM, Waller F, Scholz U, Pons-Kühnemann J, Sonnewald S, Sonnewald U, Kogel KH. Manipulation of plant innate immunity and gibberellin as factor of compatibility in the mutualistic association of barley roots with *Piriformospora indica*. The Plant Journal. 2009; 59:461-474.

^9^Deshmukh SD, Kogel KH. *Piriformospora indica* protects barley from root rot caused by *Fusarium graminearum*. Journal of Plant Disease and Protection. 2007; 114:263-268.
